# Regional anesthesia did not improve postoperative long-term survival of tumor patients: a systematic review and meta-analysis of randomized controlled trials

**DOI:** 10.1186/s12957-023-02957-3

**Published:** 2023-02-28

**Authors:** Tao Li, Xiangrui Meng, Di Wang, Qiang Wang, Jiahai Ma, Zhao Dai

**Affiliations:** 1 Department of Anesthesiology, The Affiliated Yantai Yuhuangding Hospital of Qingdao University, Yantai, 264000 Shandong China; 2 Operating Room, The Affiliated Yantai Yuhuangding Hospital of Qingdao University, Yantai, 264000 Shandong China; 3 Department of Internal medicine, Yantai Haigang Hospital, Yantai, 264000 Shandong China; 4 Department of General Surgery, Taian Municipal Hospital, Taian, 271000 Shandong China

**Keywords:** Regional anesthesia, General anesthesia, Tumor, Recurrence, Survival

## Abstract

**Objective:**

Experimental research and clinical trials have reported a positive effect of regional anesthesia (RA) on prognosis of cancers. We systematically reviewed the efficacy of RA on recurrence-free survival (RFS) and overall survival (OS) after oncology surgeries.

**Methods:**

PubMed, Cochrane library, and Embase were searched from inception to June 20, 2022 for RCTs in which any form of RA was initiated perioperatively. Time-to-event data (hazard ratio (HR)) were extracted independently and in duplicate. The primary outcome was the association of RA with RFS and OS, while the secondary outcomes included time to tumor progression, 5-year RFS, and 5-year OS.

**Results:**

Fifteen RCTs with 5981 participants were included. Compared to GA, RA has no positive effect on RFS (HR, − 0.02; 95% CI, − 0.11 to 0.07), OS (HR, − 0.03; 95% CI, − 0.28 to 0.23), time to tumor progression (0.11; 95% CI, − 0.33 to 0.55), 5-year RFS (risk ratio (RR), 1.24; 95% CI, 0.88 to 1.76)), and 5-year OS (RR, 1.11; 95% CI, 0.85 to 1.44). Subgroup analysis based on study design, patient characteristics and tumor types also showed no effect of RA on RFS or OS.

**Conclusions:**

Our results demonstrated that there is no significant evidence supporting the role of RA in improving long-term survival after oncology surgeries.

**Supplementary Information:**

The online version contains supplementary material available at 10.1186/s12957-023-02957-3.

## Introduction

For the treatment of cancer, especially for the early or middle stage, surgery is an important procedure and it is necessary in some cases. However, cancer recurrence following surgery becomes a major threat to patient’s survival. Cancer recurrence rather than the primary tumor causes about 90% deaths of patients [1]. Perioperative immunomodulation is believed to be critical to tumor recurrence [2]. During surgical procedures, there are several major factors that may influence perioperative immunomodulation and may contribute to metastatic spread, which may lead to a reduction of patients survival and prognosis [3, 4]. Through stimulating the hypothalamic-pituitary-adrenal (HPA) axis and sympathetic nervous system (SNS) responsible for the release of several neuroendocrine mediators such as vascular endothelial growth factor and matrix metalloproteinases, surgery may suppresses the host immune and activates micro metastasis [5].

The effect of anesthesia agents on tumor progression has been explored widely. Studies showed that GA with analgesia and amnesia drugs such as thiopental or volatile agents inhibited host immunity by modulating natural killer cell, macrophage, and neutrophil function [6, 7]. Using opioids for intraoperative and postoperative pain management also inhibited cell-mediated immunity (CMI) and promote tumor cell proliferation through stimulating angiogenesis [7]. Compared with GA, RA were believed to be positive for cancer prognosis [8]. Local anesthetics seem to inhibit tumor growth via several mechanisms including cytotoxicity and promotion of apoptosis; modulation of gene expression via methylation; and inhibition of proliferation, migration, and invasion [9].

The following theories supported the view above. Firstly, local anesthetic was involved in mediating tumor growth and metastasis via influencing the biological behavior of tumor cells. Local anesthetics inhibit cell proliferation by inhibiting catecholamines release [10, 11]. RA also reduces the excitability of cancer cells by blocking voltage-gated sodium channels, thereby reducing the metastatic potential [12]. Secondly, local anesthetics preserves immune function which inhibits tumor recurrence [13]. Thirdly, as opioids can promote the proliferation of tumor cells and stimulate epithelial-mesenchymal transition and promote metastasis of tumor cells [14]. RA may impact the risk of proliferation after cancer surgery by reducing pain and opioid consumption [15]. Fourthly, local anesthetics reduced systemic angiogenic factor concentration to inhibit tumor cell growth and proliferation.

Clinical trials have investigated the relationship of RA and long-term survivals, of which some showed positive effects, while others were opposite. However, most of those were observational studies. In 2019, Sessler et al. completed the first multi-center RCT of RA on tumor survivals with a large sample and high quality and then there were few multi-center and single-center RCTs concentrated on this topic published from 2019 to 2022. Therefore, the effect of RA on cancer recurrence and patient survival is a controversial issue. In view of the above results of experimental and clinical studies, this meta-analysis assess the effect of RA on postoperative long-term survivals.

## Materials and methods

This systematic review and meta-analysis was performed following the guidelines set forth in Preferred Reporting Items for Systematic Reviews and Meta-Analyses (PRISMA). The check list and flow chart is presented in Additional file 1. Objectives, inclusion criteria, and methods have been pre-specified in a review protocol (registered in PROSPERO, CRD42018110181. URL: www.crd.york.ac.uk/PROSPERO).

### Data sources and searches

We searched PubMed, Cochrane library, and Embase from inception to August 4, 2021 based on PICOS (Patients: patients undergoing tumor procedures usually provided under general and regional anesthesia; Interventions: Any type of anesthesia which is implemented during tumor procedures; Comparators: eligible control groups were general anesthesia and regional anesthesia; Outcomes: the included trials reported on at least one of the following outcomes: recurrence, metastasis, survival, prognosis, death or mortality; Study design: only randomized controlled trials were included). The full electronic search strategy based on PICOS is presented in Additional file 2. We also checked the reference lists of relevant meta-analysis and reviews for additional studies.

### Inclusion criteria and study selection

The predefined inclusion criteria were RCTs in any language with patients undergoing any oncology surgical procedure. We compared any form of RA initiated perioperatively with any form of non-RA (GA, GA plus postoperative opioids analgesia or PCA). The primary endpoint were RFS and OS. The secondary endpoints were time to tumor progression, 5-year RFS, and 5-year OS. RFS was defined as in cancer, the length of time after primary treatment for a cancer ends that the patient survives without any signs or symptoms of that cancer; OS was defined as the length of time from the date of treatment for cancer to the death date or the censored date.

### Data extraction

Two authors (Li and Meng) independently reviewed the studies and extracted data according the predesigned standard. Divergences of views were resolved through authors’ discussion or referring to a senior investigator (Dai). We extracted details of characteristics of the included studies. Hazard ratio (HR) was extracted and time-to-event data was also obtained [16]. Risk estimates with 95%CI value were extracted for analysis. HR was extracted from most of the included studies except “Finn 2017” and “Pi 2019”, of which only relative risk (RR) could be extracted. And RR from the two studies was pooled with HR, which is used as the statistics in the majority of the included studies.

### Outcomes and definitions

The primary endpoint of the meta-analysis were RFS and OS. The secondary endpoints were time to tumor progression, 5-year RFS, and 5-year OS.

### Assessment of risks of bias for the included studies

Methodological quality of the RCTs was independently assessed by two authors (Dai and Wang) according to the PRISMA recommendations [17]. The tool comprises seven sections: random sequence generation; allocation concealment; blinding of participants and personnel; blinding of outcome assessment; incomplete outcome data; and other bias. A score of “yes” (low risk of bias), “no” (high risk of bias), or “unclear” (risk of bias is unclear from the article) was assigned for each section.

### Data synthesis and analysis

The Mantel-Haenszel method with the fixed effects model were used to combine the dichotomous data, and the random variance method with the fixed effects model were used to combine the continuous data. Heterogeneity between studies was assessed via *χ*^2^*Q* statistics and *I*^2^. *P* < 0.05 or *I*^2^ > 25% was considered as existence of heterogeneity [18]. When there was significant heterogeneity among the studies (*P* > 0.05, *I*^2^ > 50%), the random effects model was applied. On the contrary, the fixed effects model was used when homogeneous outcomes were obtained (*P* < 0.05, *I*^2^ < 50%).

In the meta-analyses of the outcomes, pre-planned sensitivity analysis by removing one study each time, subgroup analysis and meta-regression analysis was used to investigate the origin of the heterogeneity. The sensitivity analysis shows whether the pooled estimate is stable or not. Compared with the primary *I*^2^ value, a smaller *I*^2^ value contributes to explain the origin of the heterogeneity. For meta-regression analysis, the magnitude of the value represents the strength of the interpretable heterogeneity and significant statistic difference was defined as a *P* value < 0.05. Funnel plot of each trial's effect size against the standard error was conducted to assess the possibility of publication bias. The presence of a publication bias was examined using funnel plots and the Egger regression test. Significant publication bias was defined as a *P* value < 0.1. The trial sequency analysis (TSA) was performed using Stata 14 with the implementation of the R 3.4.3 software, and by installing the ldbounds and metacumbounds commands. And meta-analysis was conducted using R 3.43.

## Results

### Study selection

**Fig. 1:**
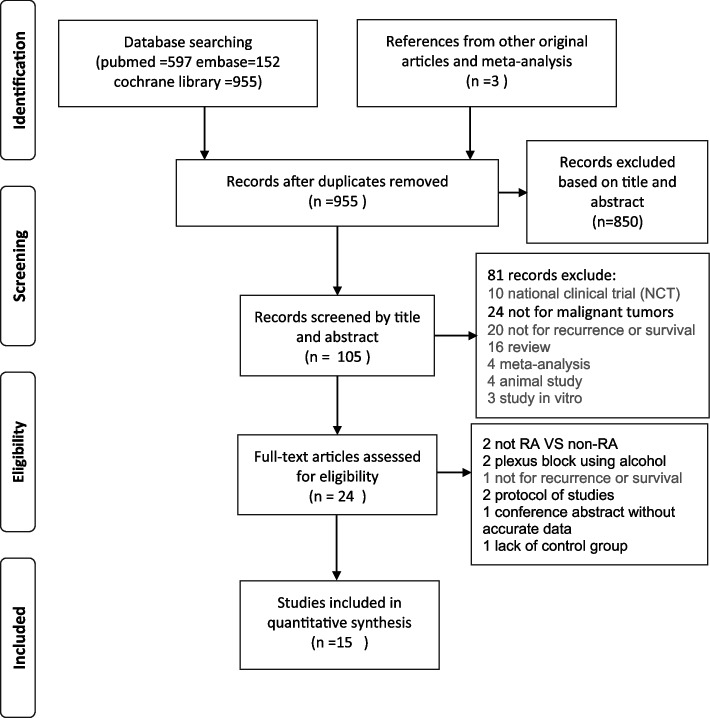
The PRISMA flow chart for literature screening

We identified 905 titles and abstracts, of which 464 articles were included after screening based on title and abstract. Followed a full-text review, 15 RCTs were finally included for analyses [19–33]. Exclusion criteria was lack of RFS or OS, not comparing RA and non-RA and plexus block using alcohol. Moreover, one study were excluded owing to unbefitting control groups. One study was published as abstract and lack of details on study variables, which was excluded (Fig. 1).

### Characteristics of studies

Characteristics of the selected studies are shown in Table 1. We included 15 RCTs and “Li 2022” was a secondary analysis of “Sessler 2019” which was not suitable for meta-analysis. RA technologies were epidural anesthesia (EDA), and nerve block. Regional analgesia was maintained from postoperative 48h to multiple days. The included studies yielded 5981 participants for analysis, of which 3007 participants received RA. Two study involved men only, 3 study involved women only (Table 1).

**Table 1:** Study characteristics

Study	Design	Tumor	Follow-up	RA (*n*)	GA (*n*)	Estimates origin	Outcome	HR (95%Cl)	Other relative outcomes
Christopherson 2008 [24]	Multi-center RCT	Colon cancer	8.3–10.8 years	GA + EDA initiated before surgery and PA until clinical appropriation (85)	GA + opioid PA (92)	Kaplan-Meier curve, log rank test	OS	0.22 (0.07–0.69)	Non-metastasis and follow-up time less than 1.46 years: RA increased OS Metastasis or beyond 1.46 years: no statistical difference
Tsui 2010 [19]	Single-center RCT	Prostate cancer	Median 4.5 years	GA + EDA intraoperatively (49)	GA (50)	Kaplan-Meier curve, log rank test	RFS	1.33 (0.64–2.77)	Median time to recurrence: HR 1.34 (0.65–2.76) Median RFS: 1644 days
Myles 2011 [25]	Multi-center RCT	Abdominal malignancies	9.0–14.8 years	GA + EDA initiated before surgery and PA for 74 h (230)	GA + PCIA (216)	Kaplan-Meier curve, log rank test	RFS OS	0.95 (0.76–1.17) 0.95 (0.77–1.18)	Median time to recurrence: HR 0.63 (0.39-1.02); Median survival time: HR 0.95 (0.77-1.18) 5-year RFS: RR 0.95 (0.78–1.15); 5-year OS: RR 0.96 (0.79-1.17)
Binczak 2013 [22]	Single-center RCT	Abdominal malignancies	Median 17.3 years	GA + EDA initiated before surgery and PA for 5 days (69)	GA + opioid analgesia (63)	Multivariate Cox model, adjusted HR	RFS OS	0.81 (0.52–1.26) 0.71 (0.47–1.07)	Median time to recurrence: 3 years vs 1.8 years 5-year RFS: 43% (32–55%) vs 24% (15–36%) 5-year OS: 51% (40–63%) vs 32% (22–44%)
Finn 2017 [20]	Single-center RCT	Breast cancer	> 2 years	GA + PVB IBS and PA for a multiple-day (26)	GA (28)	Chi-square test	RFS OS	RR 1.62 (0.29–8.91) RR 9.67 (0.55–171.23)	Not reported
Zhu 2017 [21]	Single-center RCT	Bladder cancer	3 years	GA + EDA intraoperatively (72)	GA (72)	Kaplan-Meier curve, log rank test	OS	1.17 (0.71–1.92)	RA increased survival rates of CD3+, CD4+ and CD4+/CD8+ cells during postoperative 3 days Survival rate: 1 years: 80.56% vs 83.33%; 2 years: 68.06% vs 72.22%; 3 years: 54.17% vs 59.72%
Karmakar 2017 [23]	Single-center RCT	Breast cancer	5 years	GA + single PVB (57)	GA (60)	Kaplan-Meier curve, log rank test	RFS OS	0.66 (0.11–3.97) 2.57 (0.66–9.92)	There was no difference in the risk of local cancer recurrence, metastasis or all-cause mortality between the groups (*p* = 0.79, *p* = 0.91, and *p* = 0.13)
				GA + PVB initiated before surgery and PA for 72 h (60)			RFS OS	0.79 (0.21–2.96) 1.11 (0.32–3.83)	
Sessler 2019 [26]	Multi-center RCT	Breast cancer	Median 3 years	Propofol + PVB intraoperatively (1043)	Sevoflurane + opioid (1065)	Multivariate Cox model, adjusted HR	RFS	0·97 (0·74–1·28)	Median time to recurrence: 15 vs 17 months Sensitivity analysis: China HR 0·77 (0·55–1·09) Asian ethnic origin: HR 0·78 (0.56–1.10)
Pi 2019 [29]	Single-center RCT	Lung cancer	5 years	GA + EDA intraoperatively (74)	GA (75)	Chi-square test	RFS OS	RR 1.04 (0.77–1.41) RR 1.55 (0.88–2.74)	IL-1, IL-8, hs-CRP, TNF-a, and MDA were lower in GA+EDA group (*P*<0.05), 5 year RFS: RR 1.04 (0.77–1.41); 5-year OS: RR 1.55 (0.88–2.74)
MacFater 2020 [30]	Single-center RCT	Colon cancer	8.5–9.5 years	GA + local anesthetic infusion before surgery and PA for 72 h (18)	GA (19)	Kaplan-Meier curve, log rank test	RFS OS	3.63 (0.63–20.94) 1.46 (0.53–4.03)	There was a significantly increased difference in cancer specific mortality in RA group (4) compared with the GA group (0) (*P* = 0.046).
Rangel 2021 [27]	Single-center RCT	Prostate cancer	Median 1 year	GA without opioid + TAP intraoperatively (72)	GA with opioid (71)	Kaplan-Meier curve, log rank test	Biochemical RFS	1.25 (0.62–2.52)	Time to biochemical recurrence: HR 2.82 (0.07-1.92)
Falk 2021 [28]	Multi-center RCT	Colorectal cancer	5 years	GA + EDA initiated before surgery and PA for 72 h (99)	GA + opioid analgesia (104)	Multivariate Cox model, adjusted HR	RFS	1.19 (0.61–2.31)	5-year RFS:1.19 (0.61–2.31), sensitivity analyses showed similar or somewhat lower HR, ranging from HR 1.09 to HR 1.14
Du 2021 [31]	Multi-center RCT	Abdominal malignancies	Median 5.5 years	GA + EDA initiated before surgery and PA (853)	GA + opioid analgesia (859)	Multivariate Cox model, adjusted HR	RFS OS	0.97 (0.84–1.11) 1.06 (0.91–1.24)	Cancer-specific survival: HR, 1.09 (0.93 to 1.28) Event-free survival: HR, 0.98 (0.86 to 1.12)
Xu 2021 [32]	Single-center RCT	Lung cancer	Median 3 years	GA + EDA initiated before surgery and PA for 72 h (200)	GA (200)	Multivariate Cox model, adjusted HR	RFS OS	0.90 (0.60–1.35) 1.12 (0.64–1.96)	Cancer-specific survival: HR, 1.08 (0.61–1.91)
Li 2022 [33]	“Sessler 2019” (subgroup)	The same as Sessler 2019	Median 4.4 years	The same as Sessler 2019 (624)	The same as Sessler (629)	Multivariate Cox model, adjusted HR	RFS	0.92 (0.67–1.26)	Estrogen receptor negative: HR, 0.80 (0.50–1.30) Estrogen receptor positive: HR, 1.06 (0.71–1.60)

*OS* over survival, *RFS* recurrence-free survival, *PA* postoperative analgesia, *EDA* epidural anesthesia, *PVB* paravertebral block, *TAP* transversus abdominis plane, *RA* regional anesthesia, *GA* general anesthesia, *PCEA* patient-controlled epidural analgesia, *PCIA* patient-controlled intravenous analgesia, *HR* hazard ratio, *RR* risk ratio

### Risk of bias assessment

**Fig. 2:**
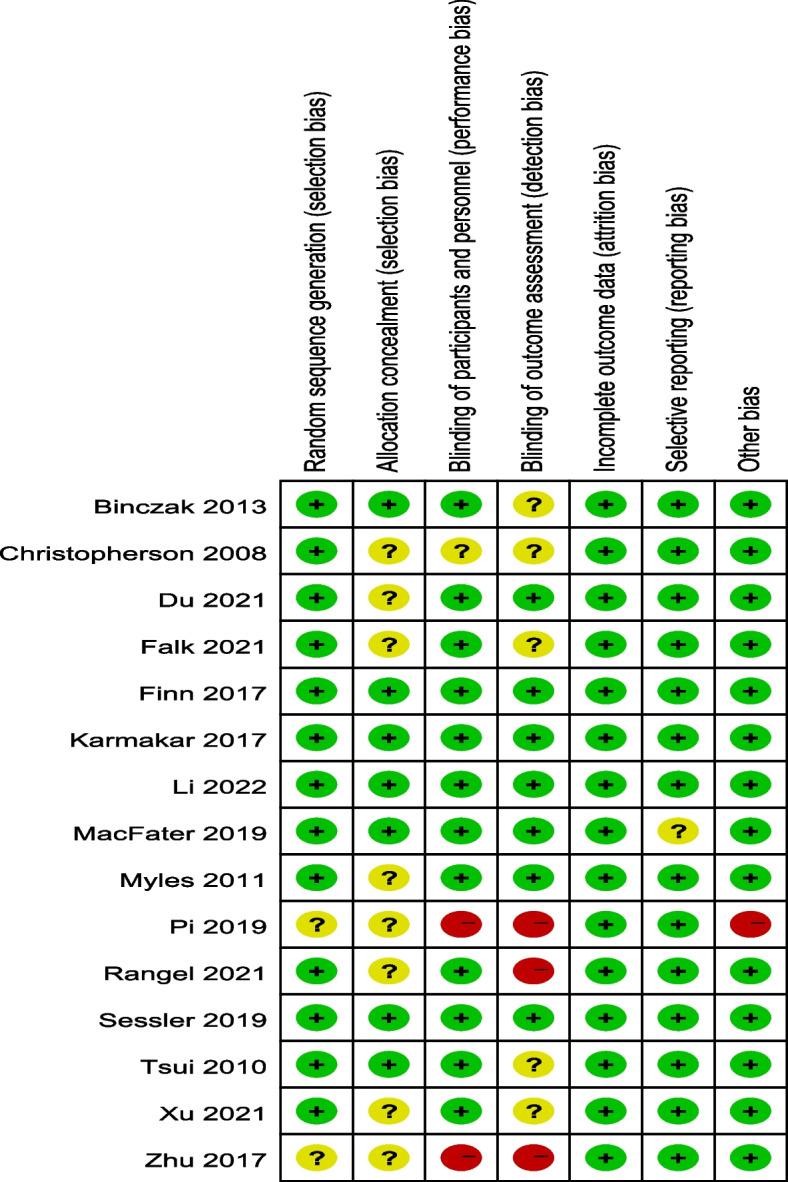
Risk of bias in individual studies

Quality of the included RCTs were presented in Fig. 2. The methodological bias of the included studies was relatively low, indicating a high qualities of the studies.

### Outcomes

#### Meta-analysis of the primary endpoint

**Fig. 3:**
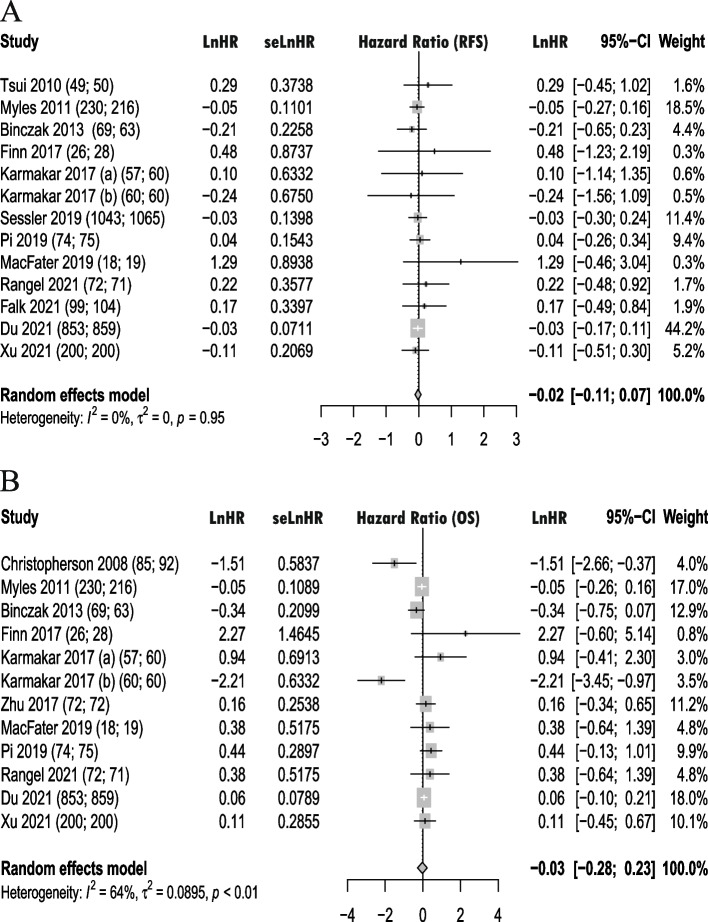
Forest plot of regional anesthesia on postoperative RFS and OS. **A** Forest plot of regional anesthesia on recurrence-free survival. **B** Forest plot of regional anesthesia on overall survival

There was no statistical difference of RA on long-term RFS and OS after oncology studies compared to GA. The pooled estimates of RFS and OS was (HR, − 0.02; 95% CI, − 0.11 to 0.07; *I*^2^, 0%) and (HR, − 0.03; 95% CI, − 0.28 to 0.23; *I*^2^, 64%), respectively (Fig. 3A, B).

##### Subgroup analysis

**Fig. 4:**
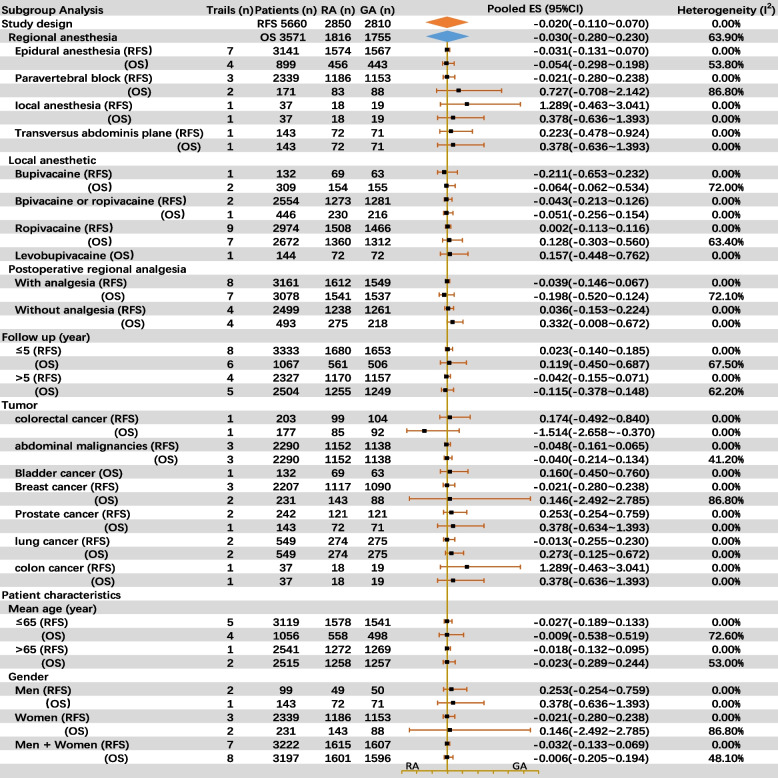
Forest plot of subgroup analysis; pooled ES, pooled estimate (LnHR)

Based on study design, patient characteristics and tumor type, 7 subgroups were established. Neither EDA nor PVB improved postoperative survivals. Whether administration of postoperative regional analgesia or not was associated with long-term survivals. Local anesthetic types were not associated with RFS or OS. RA had neither significant effect on survivals in studies with follow-up time median 5 or less years nor more than 5 years. There were no statistical difference of RA on long-term survivals for patients undergoing colorectal cancer, abdominal malignancies, bladder cancer, breast cancer, and prostate cancer. And the negative effect for RA on RFS and OS was not associated with age nor gender of the participants (Fig. 4).

##### Meta-regression

Meta-regression results were shown in Table 2. Chosen variables were not able to fully explain the low degree of heterogeneity on OS, leaving most of the heterogeneity unexplained.

**Table 2:** Meta-regression for RFS and OS

RFS	tau^2^	*R*^2^	*P* value	OS	tau^2^	*R*^2^	*P* value
Regional anesthesia	0	NA	> 0.05	Regional anesthesia	0.40	− 49.77%	> 0.05
Local anesthetic	0	NA	> 0.05	Local anesthetic	0.13	37.15%	> 0.05
Regional analgesia	0	NA	> 0.05	Regional analgesia	0.18	34.91%	> 0.05
Follow up	0	NA	> 0.05	Follow up	0.35	− 30.34%	> 0.05
Tumor	0	NA	> 0.05	Tumor	0.17	36.08%	> 0.05
Mean age	0	NA	> 0.05	Mean age	0.39	− 44.95%	> 0.05
Gender	0	NA	> 0.05	Gender	0.28	− 6.81%	> 0.05

*RFS* recurrence-free survival, *OS* overall survival, *tau*^*2*^ estimated amount of residual heterogeneity, *R*^*2*^ amount of heterogeneity accounted for, *NA* not analysis

##### Sensitivity analysis

**Fig. 5:**
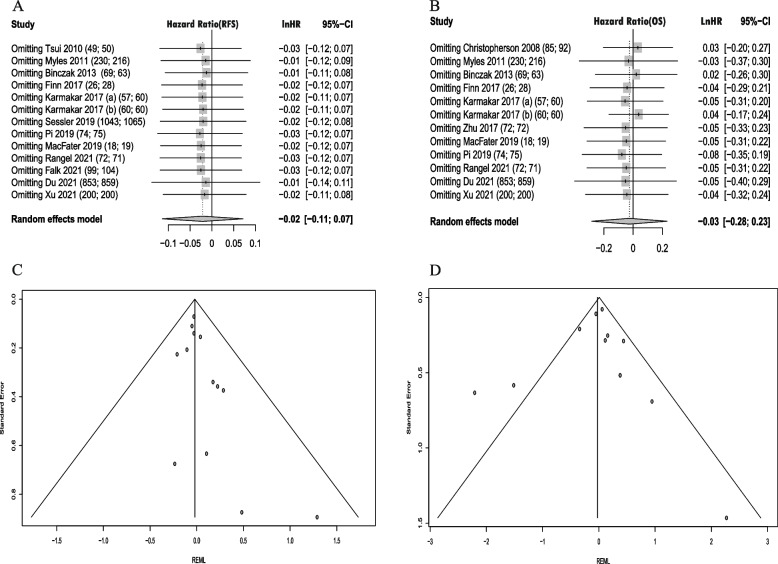
Sensitivity analysis and funnel plots for postoperative RFS and OS. **A** Sensitivity analysis of regional anesthesia on recurrence-free survival. **B** Sensitivity analysis of regional anesthesia on overall survival. **C** Funnel plot for postoperative recurrence-free survival. **D** Funnel plot for postoperative overall survival

The sensitivity analysis showed the pooled estimate and 95%CI for RFS did not change significantly followed by each study being excluded separately. And the pooled estimate and 95%CI for OS changed significantly followed by one study (Binczak 2013) being removed (Fig. 5A, B), and the *I*^2^ was 0% after the study being excluded. Followed by “Finn 2017” and “Pi 2019” (only reported RRs) being removed, the pooled estimates of RFS and OS were − 0.03 (− 0.13 to 0.07; *I*^2^, 0%) and − 0.09 (− 0.36 to 0.17; *I*^2^, 65%).

##### Publication bias

Funnel plots did not show deviations from symmetry (Fig. 5C, D). The Egger’s test indicated the funnel plots was symmetry for both RFS and OS (*P* = 0.672, *P* = 0.133) and no publication bias was found.

#### Meta-analysis of the secondary endpoints

**Fig. 6:**
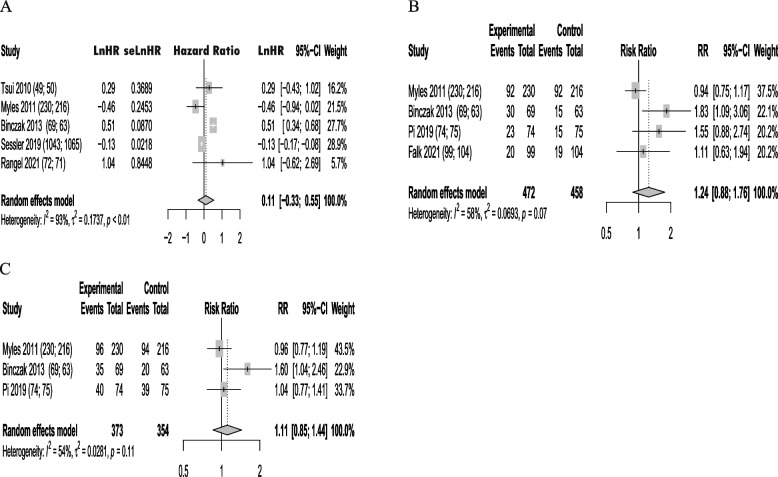
Forest plots of regional anesthesia on time to tumor progression, 5-year RFS, and 5-year OS. **A** Forest plot of regional anesthesia on time to tumor progression, HR was extracted and was pooled. **B** Forest plot of regional anesthesia on 5-year RFS. **C** Forest plot of regional anesthesia on 5-year OS

There was no statistical difference of RA on tumor progression after oncology surgeries compared to GA (HR, 0.11; 95% CI, − 0.33 to 0.55; *I*^2^, 93%) (Fig. 6A). There were also no statistical difference of RA on 5-year RFS (RR, 1.24; 95% CI, 0.88 to 1.76; I2, 58%) (Fig. 6B) and 5-year OS (RR, 1.11; 95% CI, 0.85 to 1.44; I2, 54%) (Fig. 6C).

#### Trial sequential analysis

**Fig. 7:**
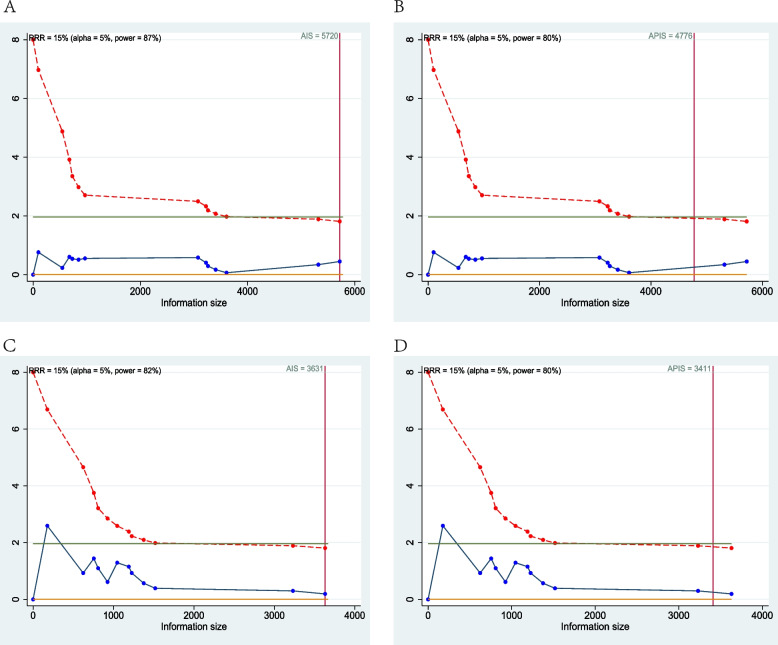
Trial sequential analysis of trials reporting HR between RA and GA on postoperative RFS and OS; green line (*Z* = 1.96), dashed red line (monitoring boundary, UB), blue line (cumulative *z* curve), red line (sample size); the cumulative *z* curve was constructed using a random-effects model. **A** The AIS graph for RFS. **B** The APIS graph for RFS. **C** The AIS graph for OS. **D** The APIS graph for OS; the APIS graph showed that for an RRR of 38%, alpha 5%, and a power of 80%, the number of optimal patients for RFS is 4776 and for OS is 3411, *z* curve (blue line) reached the information size (red line) for both RFS and OS which provides firm evidence of effect

TSA was performed to evaluate the potency of the result of the meta-analysis and estimate a sample size for RCTs, adjusting the results to avoid types I and II errors. The program used was Stata 14, with the integration of the R 3.4.3 software, through the metacumbounds commands [34]. The O’Brien–Fleming spending function was used by applying random effects. The APIS (a priori information size) and, subsequently, the AIS (accrued information size) commands were used via the dialog box to determine the optimal sample size and the power of the results, assuming an RRR (reduction risk relative) of 15%, an alpha value equal to 5% (type 1 error), and beta at 20% (type 2 error). The z curves reached the information size provided firm evidence of effect both on RFS (Fig. 7A, B) and OS (Fig. 7C, D). The APIS graph showed that for an RRR of 15%, alpha 5%, and a power of 80%, the number of optimal patients is 4776 for RFS and 3411 for OS.

## Discussion

### Summary of evidence

The results of our systematic review and meta-analysis indicated that RA had no significant effect on postoperative RFS and OS. This meta-analysis included 15 RCTs with 5981 participants. Heterogeneity analysis indicated no statistical heterogeneity of RA on RFS and a moderate amount of statistical heterogeneity on OS. We also established several clinically relevant subgroups to find different results. The subgroup analysis based on study design, patient characteristics and tumor type showed no statistic significant of RA on long-term survivals. Meta-regression was applied to investigate the origin of the moderate amount of statistical heterogeneity (*I*^2^ = 64%) on OS. As shown in Table 2, the results of meta-regression did not find the origin of heterogeneity. So, we implemented sensitivity analysis subsequently. The sensitivity analysis showed a relevant stable result and the pooled estimate and 95%CI for OS changed significantly followed by 1 study (Binczak 2013) being removed (I^2^ = 0%). As the heterogeneity is moderate and the sample size of the study is relatively small, we believe that removing this study has little effect on the result. Followed by “Finn 2017” and “Pi 2019” (only reported RRs) being removed, the pooled estimates of RFS and OS were − 0.03 (− 0.13 to 0.07; *I*^2^, 0%) and − 0.09 (− 0.36 to 0.17; I2, 65%). There is no statistical difference between the two groups on RFS and OS followed by the two studies eliminated. *I*^2^ (0% and 64%) was also similar to before (0% and 65%). So “Finn 2017” and “Pi 2019” does not pose enough interference to HRs and *I*^2^ and we believe the results of the meta-analysis was stable.

Classical theories of recurrence and progression suggest that immune function is a key factor in tumor cells survival [35, 36]. Some research investigated the association between analgesia agents and tumor progression. For example, μ-opioid receptor agonist increases tumor cell migration, growth, and metastasis [37]. In contrast, local anesthetics not only inhibits tumor cell migration pathways [38]; prevents cell differentiation or proliferation [39]; and alleviates the chronic pain and anti-inflammatory effects [40, 41], but also reduces perioperative opioids. Some clinical trials showed that RA significantly improved recurrence and slowed down the progression [42, 43], resulting in prolonged survivals [44, 45] and previous meta-analysis find a positive association between RA and OS [46]. However, the above studies were research in vitro or observational studies. Some of the previous meta-analysis only investigated one type of tumor and did not include RCTs or extracted limited time-to-event data from the article [47]. This meta-analysis included all RCTs or secondary data from previous RCTs to product accurate results and showed RA could not improve cancer RFS and OS, which were not consistent with results of previous research. We predict the following reasons for this result: (1) there are many factors influencing the recurrence and metastasis of tumor. Anesthetic agents is one factor of those in vitro. But in vivo, as the internal environment is complicated, experiment in vitro is unable to reflect the situation in the human body. (2) Observation studies are lack of randomization, blinding and with confounding factors which influences the authenticity of results.

The meta-analysis has similar findings compared to the previous systematic reviews and meta-analysis but we added new RCTs with large samples of recent years (from 2019 to 2022). So, it was decided to perform the TSA for the evaluation of the analytical power of the data and assuming an RR of 15%, the 13 included studies for RFS and 12 included studies for OS provide results with adequate statistical power.

### Limitations

Firstly, there are 5 RCTs were purpose-designed with large sample size. The other studies included have fewer than 100 patients each group and probably contribute noise. However, risk of bias evaluation showed that the risk of bias between the studies was relative low. Meanwhile, the publication bias test showed no publication bias between the included studies. So including as many RCTs as possible contributes to maximize the sample size and reduce the confidence interval. TSA make the results more convincing.

Secondly, we analyzed several primary and secondary outcomes, which could potentially lead to an increased overall type I error rate for all outcomes under investigation.

Thirdly, we did not extend our search to conference proceedings, grey literature, and clinical trial registries. We put our best efforts into a comprehensive database search and accurate study selection strategy to provide the reader with the most updated and complete systematic review of the available literature on this topic. Embase includes conference abstracts from important medical conferences, yet, we could not identify any conference proceeding that was not eventually published in a full article form.

Fourthly, the heterogeneity in different anesthesia protocols. The scheme of general anesthesia could not be unified between the studies. Although the included studies was all RCTs with a relative high quality, combinations of different drugs and application of postoperative analgesia may mask the impact of local anesthetics.

### Future prospects

A few RCTs with large sample and high quality are being investigated to prove the above results. NCT00684229 is a multi-center RCT which included 2500 participants to investigate the effect of RA on colorectal cancer recurrence; NCT01179308 is a multi-center RCT which included 1532 participants to investigate the effect of RA on lung cancer recurrence and NCT01975064 is a multi-center RCT which included 8000 participants to compare different general anesthesia with or without additional RA on breast, colon, or rectal cancer recurrence. Considering that most of the included studies were with small sample size and were single-center RCTs, we believe the pooled estimates will be relatively stable over time.

## Conclusions

Our findings indicated that perioperative RA did not reduce postoperative RFS, OS, 5-year RFS, 5-year OS, or time to tumor progress.

## Supplementary Information

**Additional file 1.** PRISMA 2009 Checklist.

**Additional file 2.** Search strategy based on PICOS.

## Data Availability

Not applicable.

## Abbreviations

RA: Regional anesthesia
GA: General anesthesia
HR: Hazard ratio
RR: Relative risk
RFS: Recurrence-free survival
OS: Overall survival
PCA: Patient-controlled analgesia
TSA: Trial sequential analysis
RRR: Reduction risk relative
