# Supplementary material for: Regional anesthesia did not improve postoperative long-term survival of tumor patients: a systematic review and meta-analysis of randomized controlled trials
Source: World J Surg Oncol. 2023 Feb 28;21:68. doi: 10.1186/s12957-023-02957-3 (PMC9972672; doi:10.1186/s12957-023-02957-3)
Supplement: Supplementary file 2 — Additional file 2. Search strategy based on PICOS. [file 12957_2023_2957_MOESM2_ESM.doc]

Appendix 2 Search strategy based on PICOS

Pubmed

1. Anesthesia, Intravenous [MeSH] OR text word: Anesthesias, Intravenous; Intravenous Anesthesia; Intravenous Anesthesias

2. Anesthesia, Local [MeSH] OR text word: Local Anesthesia; Anesthesia, Infiltration; Infiltration Anesthesia; Neural Therapy of Huneke; Huneke Neural Therapy

3. Anesthesia, Endotracheal [MeSH] OR text word: Anesthesias, Endotracheal; Endotracheal Anesthesias; Intratracheal Anesthesia; Anesthesias, Intratracheal; Intratracheal Anesthesias; Anesthesia, Intratracheal

4. Anesthesia, Inhalation [MeSH] OR text word: Inhalation Anesthesia; Insufflation Anesthesia; Anesthesia, Insufflation

5. General Anesthesia [MeSH]; text word: Anesthesias, General; General Anesthesia; General Anesthesias

6. Anesthesia, Epidural [MeSH] OR text word: Anesthesia, Peridural; Anesthesias, Peridural; Peridural Anesthesia; Peridural Anesthesias; Anesthesia, Extradural; Anesthesias, Extradural; Extradural Anesthesia; Extradural Anesthesias; Epidural Anesthesia; Anesthesias, Epidural; Epidural Anesthesias

7. Anesthesia, Conduction [MeSH] OR text word: Conduction Anesthesia; Anesthesia, Regional; Regional Anesthesia

8. Nerve Block [MeSH] OR text word: Block, Nerve; Blocks, Nerve; Nerve Blocks; Nerve Blockade; Blockade, Nerve; Blockades, Nerve; Nerve Blockades; Chemical Neurolysis; Chemical Neurolyses; Neurolyses, Chemical; Neurolysis, Chemical; Chemodenervation; Chemodenervations

9. neuraxial anesthesia OR anesthetic technique

10. Neoplasms [MeSH] OR cancer OR maliganant tumor OR malignancy

11. recurrence OR metastasis OR survival OR prognosis OR death OR mortality

12. Randomized Controlled Trial [Mesh]

13. (1 OR 2 OR 3 OR 4 OR 5 OR 6 OR 7 OR 8 OR 9) AND 10 AND 11 AND 12 AND [human]/lim

Embase

1. 'intravenous anesthesia'/exp OR 'intravenous anesthesia' OR 'local anesthesia'/exp OR 'local anesthesia' OR 'endotracheal anesthesia'/exp OR 'endotracheal anesthesia' OR 'inhalation anesthesia'/exp OR 'inhalation anesthesia' OR 'general anesthesia'/exp OR 'general anesthesia' OR 'epidural anesthesia'/exp OR 'epidural anesthesia' OR 'regional anesthesia'/exp OR 'regional anesthesia' OR 'nerve block'/exp OR 'nerve block' OR 'neuraxial anaesthesia'/exp OR 'neuraxial anaesthesia'

2. 'anesthesias, intravenous' OR 'intravenous anesthesia' OR 'intravenous anesthesias' OR 'local anesthesia' OR 'anesthesia, infiltration' OR 'infiltration anesthesia' OR 'neural therapy of huneke' OR 'huneke neural therapy' OR 'anesthesias, endotracheal' OR 'endotracheal anesthesias' OR 'intratracheal anesthesia' OR 'anesthesias, intratracheal' OR 'intratracheal anesthesias' OR 'anesthesia, intratracheal'

3. 'inhalation anesthesia' OR 'insufflation anesthesia' OR 'anesthesia, insufflation' OR 'anesthesias, general' OR 'general anesthesia' OR 'general anesthesias' OR 'anesthesia, peridural' OR 'anesthesias, peridural' OR 'peridural anesthesia' OR 'peridural anesthesias' OR 'anesthesia, extradural' OR 'anesthesias, extradural' OR 'extradural anesthesia' OR 'extradural anesthesias' OR 'epidural anesthesia' OR 'anesthesias, epidural' OR 'epidural anesthesias'

4. 'conduction anesthesia' OR 'anesthesia, regional' OR 'regional anesthesia' OR 'block, nerve' OR 'blocks, nerve' OR 'nerve blocks' OR 'nerve blockade' OR 'blockade, nerve' OR 'blockades, nerve' OR 'nerve blockades' OR 'chemical neurolysis' OR 'chemical neurolyses' OR 'neurolyses, chemical' OR 'neurolysis, chemical' OR 'chemodenervation' OR 'chemodenervations' OR 'anesthetic technique' OR 'neuraxial anesthesia'

5. 'malignant neoplasm'/exp OR 'malignant neoplasm' OR cancer OR malignancy

6. recurrence OR metastasis OR survival OR prognosis OR death OR mortality

7. 'randomized controlled trial'/exp OR 'randomized controlled trial' OR rct OR 'randomized controlled trials'

8. (1 OR 2 OR 3 OR 4) AND 5 AND 6 AND 7 AND [humans]/lim

Cochrane library

1. Anesthesia, Intravenous [MeSH] OR text word: Anesthesias, Intravenous; Intravenous Anesthesia; Intravenous Anesthesias

2. Anesthesia, Local [MeSH] OR text word: Local Anesthesia; Anesthesia, Infiltration; Infiltration Anesthesia; Neural Therapy of Huneke; Huneke Neural Therapy

3. Anesthesia, Endotracheal [MeSH] OR text word: Anesthesias, Endotracheal; Endotracheal Anesthesias; Intratracheal Anesthesia; Anesthesias, Intratracheal; Intratracheal Anesthesias; Anesthesia, Intratracheal

4. Anesthesia, Inhalation [MeSH] OR text word: Inhalation Anesthesia; Insufflation Anesthesia; Anesthesia, Insufflation

5. General Anesthesia [MeSH]; text word: Anesthesias, General; General Anesthesia; General Anesthesias

6. Anesthesia, Epidural [MeSH] OR text word: Anesthesia, Peridural; Anesthesias, Peridural; Peridural Anesthesia; Peridural Anesthesias; Anesthesia, Extradural; Anesthesias, Extradural; Extradural Anesthesia; Extradural Anesthesias; Epidural Anesthesia; Anesthesias, Epidural; Epidural Anesthesias

7. Anesthesia, Conduction [MeSH] OR text word: Conduction Anesthesia; Anesthesia, Regional; Regional Anesthesia

8. Nerve Block [MeSH] OR text word: Block, Nerve; Blocks, Nerve; Nerve Blocks; Nerve Blockade; Blockade, Nerve; Blockades, Nerve; Nerve Blockades; Chemical Neurolysis; Chemical Neurolyses; Neurolyses, Chemical; Neurolysis, Chemical; Chemodenervation; Chemodenervations

9. neuraxial anesthesia OR anesthetic technique

10. Neoplasms [MeSH] OR cancer OR maliganant tumor OR malignancy

11. recurrence OR metastasis OR survival OR prognosis OR death OR mortality

12. Randomized Controlled Trial [Mesh]

13. (1 OR 2 OR 3 OR 4 OR 5 OR 6 OR 7 OR 8 OR 9) AND 10 AND 11 AND 12
